# Supplementary material for: Homologue replacement in the import motor of the mitochondrial inner membrane of trypanosomes
Source: eLife. 2020 Feb 27;9:e52560. doi: 10.7554/eLife.52560 (PMC7064343; doi:10.7554/eLife.52560)
Supplement: Transparent reporting form [file elife-52560-transrepform.docx]

***eLife’s* transparent reporting form**

We encourage authors to provide detailed information *within their submission* to facilitate the interpretation and replication of experiments. Authors can upload supporting documentation to indicate the use of appropriate reporting guidelines for health-related research (see [EQUATOR Network](http://www.equator-network.org/%20)), life science research (see the [BioSharing Information Resource](https://biosharing.org/)), or the [ARRIVE guidelines](http://www.plosbiology.org/article/info:doi/10.1371/journal.pbio.1000412) for reporting work involving animal research. Where applicable, authors should refer to any relevant reporting standards documents in this form.

If you have any questions, please consult our Journal Policies and/or contact us: [editorial@elifesciences.org](mailto:editorial@elifesciences.org).

**Sample-size estimation**

- You should state whether an appropriate sample size was computed when the study was being designed
- You should state the statistical method of sample size computation and any required assumptions
- If no explicit power analysis was used, you should describe how you decided what sample (replicate) size (number) to use

Please outline where this information can be found within the submission (e.g., sections or figure legends), or explain why this information doesn’t apply to your submission:

State of the art SILAC immmunoprecipitation (IP) experiment with subsequent MS analysis are routinely done in triplicates (indicated in the corresponding legends)

State of the art SILAC RNAi experiments with subsequent MS analysis are routinely done in triplicates (indicated in the corresponding legends)

**Replicates**

- You should report how often each experiment was performed
- You should include a definition of biological versus technical replication
- The data obtained should be provided and sufficient information should be provided to indicate the number of independent biological and/or technical replicates
- If you encountered any outliers, you should describe how these were handled
- Criteria for exclusion/inclusion of data should be clearly stated
- High-throughput sequence data should be uploaded before submission, with a private link for reviewers provided (these are available from both GEO and ArrayExpress)

Please outline where this information can be found within the submission (e.g., sections or figure legends), or explain why this information doesn’t apply to your submission:

The SILAC IP MS analyses shown in Fig. 3B (TbPam16/TbPam18) were done in triplicates. This is indicated in the corresponding legends.

The new SILAC IP MS analysis shown in Fig. 6 – supplement 1 (ACAD) was done in triplicates. This is indicated in the corresponding legends.

The SILAC RNAi MS analysis shown Fig. 9 and Fig. 9 – figure supplement 1 was performed in triplicates. This is indicated in the corresponding legends.

**Statistical reporting**

- Statistical analysis methods should be described and justified
- Raw data should be presented in figures whenever informative to do so (typically when N per group is less than 10)
- For each experiment, you should identify the statistical tests used, exact values of N, definitions of center, methods of multiple test correction, and dispersion and precision measures (e.g., mean, median, SD, SEM, confidence intervals; and, for the major substantive results, a measure of effect size (e.g., Pearson's r, Cohen's d)
- Report exact p-values wherever possible alongside the summary statistics and 95% confidence intervals. These should be reported for all key questions and not only when the p-value is less than 0.05.

Please outline where this information can be found within the submission (e.g., sections or figure legends), or explain why this information doesn’t apply to your submission:

The statistics used for the bioinformatic analysis shown in Fig. 1, Fig. 1 - figure supplement 1, Fig. 1 - and figure supplement 2 are described in the corresponding figure legends as well as in the Material and Methods sections “Protein similarity network analysis”, “Assembly of diplomenid transcriptomes” and “Phylogenetic analysis”

The SILAC IP MS analyses shown in Fig. 3B (TbPam16/TbPam18) were done in triplicates. This is indicated in the corresponding legends and the Material and Methods section LC-MS and data analysis.

The new SILAC IP MS analysis shown in Fig. 6 – supplement 1 (ACAD) was done in triplicates. This is indicated in the corresponding legends and the Material and Methods section LC-MS and data analysis.

The standard statistical analysis applied to the SILAC RNAi MS data shown Fig. 9 and Fig. 9 – figure supplement 1 was done in triplicates. This is indicated in the corresponding legends and the Material and Methods section LC-MS and data analysis.

(For large datasets, or papers with a very large number of statistical tests, you may upload a single table file with tests, Ns, etc., with reference to sections in the manuscript.)

**Group allocation**

- Indicate how samples were allocated into experimental groups (in the case of clinical studies, please specify allocation to treatment method); if randomization was used, please also state if restricted randomization was applied
- Indicate if masking was used during group allocation, data collection and/or data analysis

Please outline where this information can be found within the submission (e.g., sections or figure legends), or explain why this information doesn’t apply to your submission:

Does not apply to neither the bioinformatic analysis nor to the pulldown experiment

**Additional data files (“source data”)**

- We encourage you to upload relevant additional data files, such as numerical data that are represented as a graph in a figure, or as a summary table
- Where provided, these should be in the most useful format, and they can be uploaded as “Source data” files linked to a main figure or table
- Include model definition files including the full list of parameters used
- Include code used for data analysis (e.g., R, MatLab)
- Avoid stating that data files are “available upon request”

Please indicate the figures or tables for which source data files have been provided:

Figure 1 is linked to Figure 1 – Source Data 1

Figure 3 is linked to Figure 3 – Source Data 1

Figure 3 is linked to Figure 3 – Source Data 2

Figure 3 is linked to Figure 3 – Source Data 3

Figure 9 is linked Figure 9 – Source Data 1
